# A Novel Signal Processing Measure to Identify Exact and Inexact Tandem Repeat Patterns in DNA Sequences

**DOI:** 10.1155/2007/43596

**Published:** 2007-03-13

**Authors:** Ravi Gupta, Divya Sarthi, Ankush Mittal, Kuldip Singh

**Affiliations:** 1 Department of Electronics and Computer Engineering, Indian Institute of Technology Roorkee, Roorkee, Uttaranchal 247 667, India

## Abstract

The identification and analysis of repetitive patterns are active areas of biological and computational research. Tandem repeats in telomeres play a role in cancer and hypervariable trinucleotide tandem repeats are linked to over a dozen major neurodegenerative genetic disorders. In this paper, we present an algorithm to identify the exact and inexact repeat patterns in DNA sequences based on orthogonal exactly periodic subspace decomposition technique. Using the new measure our algorithm resolves the problems like whether the repeat pattern is of period  or its multiple (i.e., 2, 3, etc.), and several other problems that were present in previous signal-processing-based algorithms. We present an efficient algorithm of , where  is the length of DNA sequence and  is the window length, for identifying repeats. The algorithm operates in two stages. In the first stage, each nucleotide is analyzed separately for periodicity, and in the second stage, the periodic information of each nucleotide is combined together to identify the tandem repeats. Datasets having exact and inexact repeats were taken up for the experimental purpose. The experimental result shows the effectiveness of the approach.

## 

[1,2,3,4,5,6,7,8,9,10,11,12,13,14,15,16,17,18,19,20]
